# Outcomes of Common Peroneal Nerve Decompression

**DOI:** 10.7759/cureus.68526

**Published:** 2024-09-03

**Authors:** John D King, Chukwuweike U Gwam, Natalie E Cignetti, Karanpreet K Dhaliwal, J. Benjamin Gordon, Xue Ma, Zhongyu Li

**Affiliations:** 1 Department of Orthopedic Surgery, Wake Forest University School of Medicine, Winston-Salem, USA

**Keywords:** pain, sensation, motor function, decompression, peroneal nerve

## Abstract

Purpose: Common peroneal nerve (CPN) neuropathy is the most common lower extremity mononeuropathy. When delayed or no recovery from CPN neuropathy is suspected, surgical CPN decompression (CPND) is considered to relieve symptoms. This study aimed to evaluate patient outcomes post-CPND performed by a single surgeon at a tertiary medical center.

Methods: Patient outcomes after CPND performed by a single surgeon were reviewed. Motor, sensation, and pain scores post-CPND were assessed in 47 of the 46 patients. Patient demographics, including age, concomitant morbidities, time from injury to surgery, and body mass index (BMI), were also analyzed for correlations with outcomes after CPND by logistic regression.

Results: 29/34 patients with impaired motor function improved by at least one motor grade, 19/42 with altered sensation reported restored normal sensation, and 31/37 reported improved pain after CPND. No correlation of patient demographic factors with motor or pain improvement after CPND was observed. However, a BMI greater than 29.15 and a time between injury and surgery exceeding 506 days were associated with lower odds of reporting restored sensation.

Conclusions: Operative decompression of CPN neuropathy improves objective motor scores and subjective sensation and pain scores.

## Introduction

Common peroneal nerve (CPN) neuropathy is the most prevalent lower extremity mononeuropathy [1]. The CPN is highly susceptible to injury at the knee due to its superficial course around the lateral fibular neck [2]. This superficial course makes it susceptible to compression injuries which are the most common etiology of CPN neuropathy. Other common causes of injury include postural positioning, weight loss, trauma, neoplasm, iatrogenic causes, and internal compression from fascial bands [3].  

Diagnosis of CPN neuropathy can be challenging, with misdiagnosis occurring in almost half of cases due to the incorrect assessment of inversion with slight foot dorsiflexion [4]. Damage along the nerve pathway can lead to symptoms of peroneal neuropathy; common causes of such damage in the United States include lumbar radiculopathy, lumbosacral trunk compression, and sciatic neuropathy. Diagnosis typically relies on clinical evaluation but may require confirmatory tests such as electromyography (EMG) or imaging when suspecting trauma or compression [1].  

Initial management of CPN neuropathy involves conservative management, such as physical therapy, avoidance of activities and positions leading to CPN compression, and padding of the fibular head after direct traumatic injury. In cases with foot drop and weakness, orthotic devices may also be considered [3]. Indications for CPN decompression (CPND) include cases with limited recovery or deterioration after 3-6 months of conservative management. Open wounds with associated CPN neuropathy symptoms should be considered an emergency and undergo exploratory surgery within 72 hours [3,5]. Early exploration has also been advocated at three months for CPN neuropathy after total knee arthroplasty (TKA) [6,7].  

In this study, we found that operative CPND improves functional recovery on motor, sensation, and pain scores.

## Materials and methods

A retrospective study on the outcomes of CPND surgery was approved by the Advocate Health-Wake Forest University School of Medicine Institutional Review Board (approval number: IRB00051286). The electronic medical records from a tertiary medical center for patients who underwent CPND surgery between October 2012 and April 2021 were reviewed. Cases involving surgical revisions, patients under 18 years old, and those requiring nerve repair or grafting were excluded. This left 47 cases involving 46 patients including 27 males and 19 females that met our inclusion criteria. The demographics of the selected patients were summarized in Table 1.  Motor outcomes were assessed using the Medical Research Council (MRC) grading scale for the tibialis anterior (TA), peroneus muscle, extensor hallucis longus (EHL), and extensor digitorum longus (EDL) using a 0-5 scale defining improvement as a one clinical grade advancement. EMG was also performed on these muscles and reported as normal or abnormal before and after surgery. Sensory outcomes were based on reports of normal sensation restoration. Tinel's sign was recorded as positive or negative before operation. Pain outcomes were evaluated using a 10-point visual analogue scale. The presence of ankle-foot orthosis (AFO) was also documented before surgery. Unfavorable outcomes were defined as no change or worsening symptoms post-operatively. Pre-operative and post-operative data were compared in patients who were followed for a minimum of six weeks and attended post-operative visits at six weeks, three months, six months, one year, and subsequent longer follow-up up to two years. The outcomes of motor function, sensation, and pain scores were summarized in Table 2 by type of injury mechanism.

**Table 1:** Summary of demographics of 47 cases of 46 patients BMI: body mass index; EMG: electromyography; CPND: common peroneal nerve decompression; CRPS: complex regional pain syndrome

Metric	Value
Mean age (years)	50.53
Age range (years)	16-79
Mean BMI	29.46 kg/m^2^
BMI range	18.87-45.77 kg/m^2^
Female	19
Male	27
Black	6
White	40
Right leg	16
Left leg	31
Abnormal EMG	34
Normal EMG	3
EMG not performed/recorded	10
Positive pre-operative Tinel's sign	34
Negative pre-operative Tinel's sign	4
Pre-operative Tinel's sign not performed/recorded	9
Mean symptom start to surgery (days)	501.51
Symptom start to surgery range (days)	4-4,551
Mean follow-up time (days)	195.62
Follow-up time range (days)	24-626
Proportion with improved motor function post-CPND	29/34
Proportion with the restoration of normal sensation post-CPND	19/42
Proportion with improved pain post-CPND	31/37
Concomitant lumbar and/or spine issues	14 patients
Concomitant CRPS	2 patients
Workers' compensation cases	7 patients
Tobacco use current or former/never	24/23 patients
Diabetic patients	3 patients, 2 with diabetic neuropathy

**Table 2:** Complaint outcomes by type of injury mechanism

	Improved motor function	Unimproved motor function	No motor function complaint	Proportion with improved motor function	Restoration of normal sensation	No restoration of normal sensation	No loss of sensation complaints	Proportion with restored normal sensation	Improved pain	Unimproved pain	No pain complaints	Proportion with improved pain
Idiopathic (11 cases, 11 patients)	7	0	4	7/7	5	2	4	5/7	10	0	1	10/10
High- and low-energy trauma/ground-level fall (20 cases, 19 patients)	13	3	4	13/16	8	11	1	8/19	9	2	9	9/11
Iatrogenic (11 cases, 11 patients)	6	1	4	6/7	5	6	0	5/11	7	4	0	7/11
Pathologic/ganglion (5 patients, 5 cases)	3	1	1	3/4	1	4	0	1/5	5	0	0	5/5

Surgical technique 

Surgical decompression was performed with loupe magnification under general anesthesia with a tourniquet. The patient was placed in the supine position, and a curvilinear incision was made laterally over the peroneal nerve and fibular neck and extended proximally and distally to expose the deep fascia. The CPN was released into the popliteal fossa and proximally dissected between the biceps and the lateral head of the gastrocnemius muscle. The superficial peroneus fascia was freed carefully over the peroneal nerve with blunt dissection. Then, following the peroneal nerve distally, the peroneal nerve was separated and released from any potential scar tissue. The fascia over the peroneus longus and brevis were released, and part of the peroneus longus was divided and retracted to expose the fibular neck. Tight deep fascia underneath the undersurface of the peroneus longus muscle was released. The superficial peroneal nerve was also released distally until the surgeon's finger was easily allowed to pass through the interval without any compression after the release, with the same procedure being performed for the deep peroneal nerve. After tourniquet release and adequate hemostasis, the wound was irrigated and closed in layers, and soft tissue dressings were placed. The patient was made weightbearing as tolerated post-operatively.  

Statistics 

Logistic regression was used to analyze patient demographics, including age, documented lumbar and cervical issues, time from injury/symptom onset to surgery, tobacco usage, chronic regional pain syndrome status, body mass index (BMI), diabetes status, workers' compensation status, sex, race, laterality, Tinel's sign presence at presentation, abnormal EMG studies, and AFO. The forward selection stepwise conditional logistic regression identified predictive variables for optimal model fit. A p-value of 0.05 or less indicated a statistically significant association with a favorable outcome. 

## Results

A single surgeon performed 47 decompressions on 46 eligible patients between 2012 and 2021. Patients had an average age of 50.53 years at surgery, with a mean symptom onset-to-surgery time of 16.5 months and a mean follow-up of 6.4 months. There were 27 males and 19 females, with an average BMI of 29.5. Pre-operative electrodiagnostic studies revealed abnormalities in 92% (34/37) of patients. Around 87% (34/39) of patients exhibited a positive Tinel's sign. Lumbar and/or cervical spine pathologies were noted in 30% (14/46) of patients, two had prior chronic regional pain syndrome complications, and three had diabetes, with two having diabetic neuropathy (DN). Seven cases involved workers' compensation (Table 1). There were two post-operative complications of cellulitis, one of which required admission with irrigation and debridement.

Regarding outcomes, 85% (29/34) of lower extremities with impaired motor function improved, 45% (19/42) with altered sensation reported restored sensation, and 84% (31/37) experienced pain improvement (Table 2).

Idiopathic

A subgroup descriptive analysis was conducted based on the etiology of the peroneal nerve injury (Table 2). In the idiopathic subgroup, all patients (7/7) with impaired motor function demonstrated improvement. Additionally, 71% (5/7) of those with altered sensation experienced a restoration of normal sensation, and every patient (10/10) with pain complaints reported an improvement in pain. 

The idiopathic group included three diabetic patients, two of whom had been diagnosed with DN. One DN patient presented with pre-operative abnormal sensation, pain, an inability to dorsiflex the ankle and great toe, and limited ankle eversion. Following CPND, this patient achieved full dorsiflexion of the ankle and toe, restored ankle eversion, regained normal sensation, and experienced the elimination of pain. The other DN patient reported only pain, which was reduced after surgery. The third diabetic patient exhibited motor weakness in ankle and toe dorsiflexion, abnormal sensation, and pain. While motor function was restored and pain reduced, normal sensation had not returned by the last follow-up visit. 

This group also included six patients who were current tobacco users and three patients who identified as former tobacco users. There were also seven patients with documented coexisting lumbar and/or cervical issues (Table 2).

Trauma

Patients were placed in the trauma group if their mechanism of injury involved low-energy injuries such as ground-level falls, ankle sprains, or low-energy fractures or high-energy injuries such as motor vehicle injuries, polytraumas, or sports-related injuries causing multi-ligamentous knee or hip dislocations. Of the 20 cases, 81% (13/16) had improved motor function, 42% (8/19) reported the restoration of normal sensation, and 82% (9/11) reported improved pain after surgical intervention (Table 2).

Iatrogenic

There were 11 cases referred to the operative surgeon for iatrogenic-related CPN, including referrals for patients following total hip arthroplasty and TKA procedures and sports medicine procedures. Of these cases, 86% (6/7) had improved motor function, 45% (5/11) had regained normal sensation, and 64% (7/11) had improved pain (Table 2). 

Ganglion

Five cases appeared to be related to an intraneural or extraneural ganglion. Of these cases, 75% (3/4) reported improved motor strength, 20% (1/5) regained normal sensation, and all five reported improved pain scores after surgical intervention (Table 2).

Patient demographics

**Figure 1:**
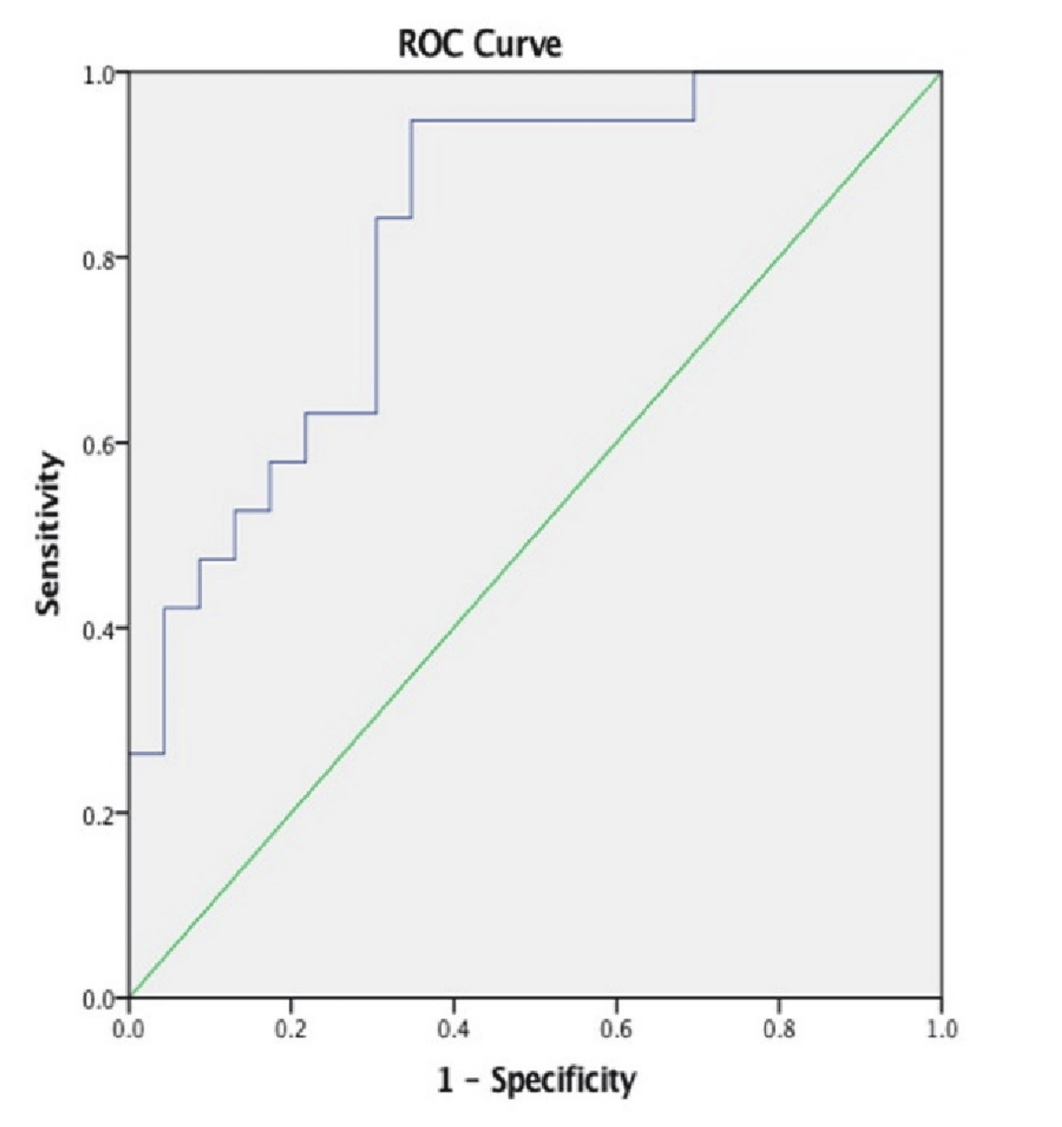
ROC curve Forward selection stepwise conditional logistic regression model demonstrated good discriminatory capacity with an area under the ROC curve of 0.8284 (0.699-0.948) ROC: receiver operating characteristic

Logistic regression identified predictors for improved motor function, improved sensation, and improved pain after CPND. There were no identifiable predictors of improved motor function or pain that could be ascertained from our data. However, a BMI greater than 29.15 (AOR: 0.845; 95% CI: 0.731-0.977; p-value=0.023), a time between injury and surgery exceeding 506 days (AOR: 1.003; 95% CI: 1.000-1.005; p-value=0.049), and workers' compensation (a trend toward statistical significance; AOR: 0.029; 95% CI: 0.000-1.950; p-value=0.099) were associated with decreased odds of reporting restored sensation (Table 3). This model demonstrated good discriminatory capacity, with an area under the receiver operating characteristic curve of 0.8284 (0.699-0.948) (Figure 1).

**Table 3:** Logistic regression model of factors associated with improved sensation BMI: body mass index

Variables	Odds ratio	Significance	95% CI
BMI	0.845	0.023	0.731-0.977
Workers' compensation	0.029	0.099	0.000-1.950
Time between injury and surgery	1.003	0.049	1.000-1.005
Smoker	4.53	0.189	0.475-43.175
Black vs. White patient	8.667	0.16	0.426-176.123
High-energy trauma	0.362	0.393	0.035-3.732
Iatrogenic	0.552	0.606	0.058-5.281
Idiopathic	3.246	0.421	0.185-57.004
Pathologic	0.134	0.193	0.006-2.764

Patient deficit profile and complaint recovery

The final recovery time point was determined as the last recorded post-op visit or when treatment was deemed successful. Most lower extremities reported complaints regarding motor function, sensation, and pain. Eight out of 25 extremities showed improvement in all three categories. The second most common presentation was motor and sensation abnormalities, with four out of eight extremities showing improvement in both. The third most common complaint profile involved sensation and pain, with three out of 18 extremities experiencing improvement. Four out of four isolated pain complaints reported pain reduction. Twenty-seven isolated sensation issues and 26 motor with pain issues were also observed, with no patients presenting with isolated motor deficits.

## Discussion

In this study, CPND demonstrated significant improvements in motor, sensation, and pain scores, consistent with findings from previous literature [1,2,8-10]. Our findings from the retrospective review observed 85% motor improvement, 45% normal sensation restoration, and 84% improved pain among 47 procedures (Table 2). These findings align with those of Humphreys et al.'s retrospective analysis, which revealed 83% motor improvement, 45% sensory restoration, and 84% pain alleviation among 51 decompressions [8]. Similarly, Ramanan and Chandran's retrospective findings revealed 74% motor improvement and 47% sensory recovery [11].

The most favorable outcomes were observed in idiopathic cases. Within the idiopathic group, all patients experienced improved motor and pain complaints, and 71% reported regained normal sensation. This finding is consistent with Maalla et al.'s study, in which 90% of idiopathic CPN neuropathy syndromes had excellent or good outcomes [2]. Idiopathic cases have been theorized to be caused by the nerve stretching over the tendinous musculature. Chronic irritation can restrict the nerve as it glides during the flexion and extension of the knee and ankle, causing scarring and adherences [12]. No associations were found between post-operative outcomes and documented lumbar/cervical issues in this group.  

In diabetic patients, all three patients experienced improvements in motor strength and pain reduction with one of the two having reported restored sensation, which aligns with current literature regarding pain reduction in diabetic patients undergoing CPND [13]. It is theorized that diabetic pathophysiology contributes to chronic compression and that peripheral nerve decompression can lead to improved diabetic symptoms and complications [9,14]. Interestingly, all three diabetic patients had coexisting lumbar and/or cervical pathologies, highlighting the possible involvement of double crush, rendering the peripheral nerve more susceptible to compression [15].  

Trauma-related CPN cases generally exhibited favorable motor recovery (81%) but less sensory improvement (42%). Most patients experienced pain relief after surgery (82%) (Table 2). These findings were consistent with reports of inferior outcomes in non-idiopathic cases of CPN neuropathy, including trauma or iatrogenic cases [1,3]. Intriguingly, here, we found that high-energy trauma patients recovered comparably to low-energy trauma patients despite the likelihood of more severe injury, possibly due to the exclusion of high-energy trauma patients who underwent peroneal nerve repair or grafting [1,8]. 

The iatrogenic subgroup, including total hip arthroplasty and TKA, showed varied outcomes but generally improved in motor function (86%), sensation (45%), and pain (64%) in this study. It should be noted that around 50% of these patients had previously undiagnosed neuropathy underlying the nerve susceptibility for stretch traction that is associated with strenuous sports activity with concomitant ligamentous injury [3]. Krackow et al. advocated for early exploration after TKA, as future recovery was noted to be unpredictable. They achieved peroneal recovery in 4/5 TKA patients and achieved similar results in patients with peroneal compromise after proximal tibial osteotomy and distal femoral osteotomy [6]. Zywiel et al. reported "peroneal dysfunction" after TKA in which patients had difficulty regaining acceptable knee range of motion and experienced transient paresthesia and dysesthesia of the lateral leg which improved following surgical decompression [16].  

Patient demographic factors did not correlate with motor or pain improvement post-CPND. Logistic regression revealed that a higher BMI (>29.15) was associated with lower odds of sensory restoration (AOR: 0.845; 95% CI: 0.731-0.977; p-value=0.023). Previous literature comparing CPND outcomes and BMI is conflicting. Humphrey et al. found that patients with BMI >25 had more unfavorable post-operative sensory outcomes than those who had a lower BMI; they did not find a relationship between BMI and post-operative motor function, similar to the findings of this study [17]. Franco et al. concluded using linear regression analysis that higher BMI was associated with decreased pain relief post-CPND [9]. A more recent meta-analysis by Wilson et al. found no significant association between BMI and post-CPND outcomes [10]. Interestingly, smoking status was found to have no association with our recovery parameters. A study by Wilson et al. however noted poorer outcomes regarding pain alleviation in both current and former smokers which they hypothesized to be due to smoking's effect on electrophysiological effects on peripheral nerves [9]. One possible reason for the discrepancies among the studies could be attributed to the small sample size of the current study to discern a significant association regarding tobacco use. Nonetheless, the finding of sensory correlation to BMI indicates that treatment plans and shared decision-making should include discussions about BMI in post-operative workups with these patients. 

Timing of surgery beyond 506 days from injury correlated with reduced sensory recovery (AOR: 1.003; 95% CI: 1.000-1.005; p-value=0.04). Current literature revealed similar reports of improved recovery with decreased time to surgery [1,2,18]. Furthermore, Maalla et al. reported a similar decrease in outcomes in patients with symptoms for more than 12 months and recommended surgery within three to four months of symptom presentation if no spontaneous improvement is noted [2]. This study observed an average of 16.5 months between symptom onset and surgery. The delay of surgery time in this study could have been due to tertiary referral center practices. Other retrospective studies also showed similar periods with month-long averages of 16 [8], 15 [11], and 28.3 months [18]. 

Workers' compensation showed a trend toward decreased odds of sensory restoration (AOR: 0.029; 95% CI: 0.000-1.950; p-value=0.099). While this CI does include zero, workers' compensation should still be considered during patient assessment and care. This model demonstrated good discriminatory capacity, with an area under the receiver operating characteristic curve of 0.771 (Figure 1). It has been well documented that workers' compensation involvement is associated with unsatisfied recovery to up to twofold greater risk of a negative outcome, which should be considered when discussing patient expectations and recovery status [19].  

Age did not have any significant association with recovery, which is consistent with previous literature [10,11]. This study included only adult patients; however, our results suggest that older patients should not be excluded for CPND.  

This study's patient recovery time endpoint was decided by the surgeon either after the patient's full recovery or the last time the patient was seen and examined before being lost to follow-up. It is important to note that some patients may have had continued or halted improvement in the post-operative period and chose not to return to the clinic. Some patients may continue to improve beyond 1.5 years after the surgical intervention. The most common presenting extremity complaint profile (25 extremities) involved all three deficits of motor dysfunction, altered sensation, and pain. Based on this study, factoring in a patient's complaint profile in align with an expectant time for a full recovery clinically, only 32% (8/25) of extremities with issues in motor, sensation, and pain had recovery in all three deficits. The second most common complaint presentation involved motor and sensation, and 50% (4/8) of extremities had recovery in both. The last extremity showed recovery by 1.5 years after surgery. Breaking down by mechanisms of injury, a clinician can use these results along with other evidence as a guide for counseling patients on when to expect recovery after surgical release. 

Limitations to this study include its retrospective design, lack of control groups, and reliance on a single surgeon for exams and operations. The exclusion of alcohol consumption is notable, given its link to polyneuropathy and poorer outcomes, with studies suggesting that alcohol consumption is a contraindication to CPND [2,20]. The study's sensory outcomes relied on subjective patient-reported restoration, while motor scores were objective, highlighting a potential bias. Additionally, in terms of suitable follow-up, we attempted to follow the patient until complete neurological recovery; however, if the patient was lost to follow-up, his or her last appointment motor, sensory, and pain scores were used as the final visit, which potentially overlooks future changes in outcomes.

## Conclusions

This study demonstrates that CPND improves impaired motor function, restores normal sensation, and reduces pain. We reported beneficial outcomes in diabetic patients, including those with DN.  

Patients with a BMI greater than 29.15 and time between injury and surgery exceeding 506 days were associated with lower odds of reporting restored sensation. Workers' compensation patients have potentially decreased odds of reporting restored sensation. These factors should be considered when discussing care and planned recovery. This study did not find any evidence of tobacco usage, age, sex, and laterality associated with recovery after CPND.  
